# MALDI-MSI Towards Multimodal Imaging: Challenges and Perspectives

**DOI:** 10.3389/fchem.2022.904688

**Published:** 2022-05-09

**Authors:** Michael Tuck, Florent Grélard, Landry Blanc, Nicolas Desbenoit

**Affiliations:** Univ. Bordeaux, CNRS, CBMN, UMR 5248, Pessac, France

**Keywords:** MALDI mass spectrometry imaging, multimodal imaging, analytical strategy, computational strategy, biological applications

## Abstract

Multimodal imaging is a powerful strategy for combining information from multiple images. It involves several fields in the acquisition, processing and interpretation of images. As multimodal imaging is a vast subject area with various combinations of imaging techniques, it has been extensively reviewed. Here we focus on Matrix-assisted Laser Desorption Ionization Mass Spectrometry Imaging (MALDI-MSI) coupling other imaging modalities in multimodal approaches. While MALDI-MS images convey a substantial amount of chemical information, they are not readily informative about the morphological nature of the tissue. By providing a supplementary modality, MALDI-MS images can be more informative and better reflect the nature of the tissue. In this mini review, we emphasize the analytical and computational strategies to address multimodal MALDI-MSI.

## Introduction

Matrix-assisted Laser Desorption Ionization Mass Spectrometry Imaging (MALDI-MSI) has established itself as one of the most attractive *ex vivo* techniques for the spatial characterization of molecules (Science, 2021). Marked by a steady stream of incremental improvements over the last 30 years, MALDI-MSI has become a premiere tool for biomedical researchers. It has been applied to numerous fields of study, from pharmacokinetics, to tumor detection and even sub-cellular metabolomics. MALDI-MSI excels in chemical specificity without prior molecular tagging, however it requires specific sample preparation and its spatial resolving capabilities relative to microscopy and even other MSI techniques are considered low. This unique set of compromises has pushed the field to combine MALDI-MSI with other imaging modalities (Masyuko et al., 2013; Matsumoto et al., 2021; Tuck et al., 2021; Lauwerends et al., 2022), whether they be other MSI techniques, other multidimensional modalities like vibrational spectroscopy, high spatial resolution imaging such as immunofluorescence microscopy (IF) or Imaging mass cytometry (IMC) (Hoch et al., 2022), and even *in vivo* techniques commonly seen in the clinic like magnetic resonance imaging (MRI). Any of these imaging techniques can supplement the information given by MALDI-MSI and alleviate its limitations. The goal is usually to perform the joint statistical analysis of the combined images, establishing spatial correlations between them. Complementary images convey different types of information, processing workflows are often suited to specific needs, with limited generalizability. The multimodal integration can be performed by various computational methods, and it is challenging to make an educated choice. Here, we attempt to review the advancements in MALDI-MSI and how they pertain to the nascent field of multimodal imaging. We classify computational methods so as to facilitate decision-making at the data processing stage.

## MALDI-MSI

MALDI-MSI has the ability to simultaneously detect thousands of ions in a single acquisition *in situ*, including proteins, peptides, glycans/polysaccharides, lipids, metabolites and pharmaceuticals (Buchberger et al., 2018). The multiplexed nature of the technique is afforded without tagging or prior knowledge of the tissue and with a mild sample preparation. Time-of-flight secondary ion MS (TOF-SIMS, Massonnet and Heeren (2019), laser ablation inductively-coupled plasma (LA-ICP, Perry et al. (2020b)), desorption electrospray ionization (DESI, Soudah et al. (2021) are other MS imaging techniques, however MALDI-MSI has emerged as the most popular as it strikes a favorable balance between sample preparation, chemical specificity/sensitivity and spatial resolution. Since its inception (Hillenkamp et al., 1975; Spengler and Hubert, 1994; Caprioli et al., 1997), MALDI-MSI has advanced incrementally in spatial resolution (Heiles et al., 2020), acquisition time (Prentice and Caprioli, 2016), and mass resolution (Spraggins et al., 2016). And yet, despite its continued amelioration, it remains shy of its potential as a molecular microscope, articulated in its earliest days (Caprioli, 2014).

### Sample Preparation

MALDI-MSI involves a simple but carefully considered sample preparation, particularly in multimodal experiments. Tissue sections 10–20 µm thick are thaw-mounted to a suitable substrate, typically a glass/metal slide. The surface of the sample should be flat to ensure optimal laser focus, yet a height profile can be used to account for topographical changes. (Bartels et al., 2017). A chemical matrix is then applied. Matrix choice and application is always a compromise between preferred analyte classes, their extraction and potential delocalization. The matrix might affect the ionization mode, adducts seen, and size of detectable analytes (Yang et al., 2018). Matrix is commonly applied by spraying or sublimation, which might affect spatial resolution *via* crystal size, analyte extraction/delocalization and reproducibility. Matrices have been thoroughly interrogated (Perry et al., 2020a) and application methods reviewed (Calvano et al., 2018).

### Quantitation

MALDI is widely viewed as qualitative. Indeed, the intensities of two MALDI-MS images cannot be directly compared because of the physics of ionization and the nature of complex tissue: the ionization efficiencies for different analyte classes differ and the signal can be suppressed due to different (biological) matrix effects (Unsihuay et al., 2021). Many challenges are not unique to MALDI-MSI; Kertesz and Cahill (2021) review quantitation of various MSI technologies.

Researchers have devised increasingly diligent approaches towards quantitative MSI (qMSI). This includes normalization/in-solution strategies like externally-sprayed standards as reference peaks or tissue extinction coefficient (TEC) (Hamm et al., 2012; Dilmetz et al., 2021), and computational strategies such as “virtual calibration” (Song et al., 2019). Also, in experimental approaches, calibration curves are employed to correlate signal intensity to known concentration of standards. Calibration curves in mimetic tissue, though more laborious, have seen good agreement with liquid chromatography-MS (Barry et al., 2019). The thoroughness of the mimetic model has made it attractive to many qMSI groups, particularly in the small molecule realm.

### MALDI-MSI Advancements

MALDI-MSI was conceived on TOF instrumentation but has since benefited from high mass resolving power provided by Fourier transform mass spectrometry (FTMS). Higher resolving power results in better analyte annotation, more peaks detected and less chemical matrix interference (Bowman et al., 2020). The addition of ion mobility has been a major advancement of MALDI-MSI as this enables the separation of isobaric species (Spraggins et al., 2019; Soltwisch et al., 2020).

Spatial resolution is another front for MALDI-MSI advancement. Alterations in laser geometry pushed the bounds of possibility, with commercial sources now capable of 10 µm spatial resolution that can often be tuned further to near one micron (Kompauer et al., 2016). Even subcellular resolutions have been achieved with transmission geometry (Zavalin et al., 2012). The spatial resolution of MALDI-MS images depends on the laser diameter and the stage step-size. It can be assessed precisely by determining the size and shape of the laser ablation marks on a grid with a resolution pattern (Fagerer et al., 2015).

Increased spatial resolution comes at the expense of sensitivity. MALDI-2 was developed to address such concerns (Soltwisch et al., 2015). Here, a secondary laser is directed at the MALDI plume and pulsed after an initial MALDI event. This ionizes neutrals and can increase sensitivity 100-fold for some molecular species (Barré et al., 2019; Soltwisch et al., 2020). This technology sparked renewed interests in transmission geometry, where researchers recently obtained ultra-high spatial resolutions, down to 600nm, with informative sensitivity by employing MALDI-2 (Niehaus et al., 2019; Spivey et al., 2019). MALDI-2 has also been combined with ion mobility (Soltwisch et al., 2020).

### Data Preprocessing and Visualization

MALDI-MS images are large datasets and enclose complex data. Various approaches are designed to reduce their complexity. For instance, peak picking and alignment reduce the dataset size while preserving relevant information along the spectral dimension (Alexandrov, 2012). As for their visualization, there are multiple commercial and open-source software available (Weiskirchen et al., 2019), many of which handle the standardized imzML data format (Römpp et al., 2011; Schramm et al., 2012).

Spectral visualization strategies, such as Kendrick mass defect analysis, have also been used to deconvolute imaging analysis (Kune et al., 2019; Blanc et al., 2021). These efforts confront a major challenge in MALDI-MSI: a lack of chemical separation.

### Data Analysis

Data analysis involves methods which facilitate the interpretation and comparison of images. Method choice depends on the experimental design, the nature of the signal, and the scientific goal of the experiment.

Dimension reduction techniques, such as principal component analysis (PCA, Trindade et al. (2018a)), non-negative matrix factorization (NMF, Trindade et al. (2018b)) or t-distributed stochastic neighbor embedding (t-SNE, van der Maaten and Hinton (2008)) can further abate the data. PCA and NMF are linear matrix decomposition techniques, whereas t-SNE is a probabilistic, non-linear dimension reduction technique. PCA and NMF decompose the original, large, MALDI-MS image into two smaller matrices, whose product approximates the original image. Common global information shared across ion images are stored in so-called component images. Component images usually highlight important structures in the tissue. PCA loadings are weights that attribute more or less importance to ion images with each component image, enacting the coupling of spatial and spectral information. NMF differs from PCA in that it imposes a non-negativity constraint on the components, making them easier to interpret. By contrast to PCA and NMF, t-SNE retains local structures by preserving distances between points in a lower-dimensional embedding. This reduction makes for visualizations that are faithful to the original image data. However, the resulting mapping is different across multiple runs and requires larger computational resources than PCA. Abdelmoula et al. (2018) use a variant of t-SNE, called hierarchical SNE (Pezzotti et al., 2016), to visualize structures with different levels of details across ion images.

Clustering methods give simplified representations of the datasets, where similar spectra are grouped together. The output can be visualized as a 2D image where each pixel has an intensity equal to its cluster number. Similarities between spectra can be estimated by various metrics, such as the Euclidean distance, which captures similarities among raw intensities, or the cosine distance, which captures similar trends in the spectra. The k-means algorithm groups spectra according to a predefined number of clusters (Palmer et al., 2015), whereas hierarchical clustering yields a tree of subregions, arranged from broad structures to finer details (Urbini et al., 2017). Alexandrov and Kobarg (2011) proposed a clustering algorithm which limits the impact of noise in MALDI-MS images, where pixels are grouped by the similarities between their neighborhoods.

Supervised machine learning methods, i.e., methods that use annotated datasets, can recognize co-localized ions from manually annotated images. Ovchinnikova et al. (2020) found spatial correlations between ion images with a deep learning model using visual similarities.

Univariate analysis can be used to find differently-abundant ions across conditions, such as different regions in the images or different samples. T-test is used to highlight statistical differences for a specific *m/z* between two different samples (Yajima et al., 2018), or regions within a tissue section, whereas the analysis of variance (ANOVA) test can be used to compare three or more regions (Blanc et al., 2018). When the normality assumption is not verified, typical in MSI datasets, non-parametric tests, such as Mann-Whitney U-test or Wilcoxon signed-rank tests can be used (Guo et al., 2014). To determine if an ion is a biomarker for a condition, Receiver Operating Characteristic (ROC) curve analysis can be applied (Hoo et al., 2017).

Data analysis usually returns several *m/z* features of interest. Metabolite databases can be polled to infer their chemical structure. The choice of the database depends on the type of analyte (e.g., LipidMaps for lipids, UniProt for proteins, CSDB for glycans, etc.). A website dedicated to MSI called METASPACE2020 allows for the interrogation of different databases.

## Multimodal MALDI-MSI

Balanced by optimism in MALDI-MSI and caution with its limitations, researchers have turned to other imaging modalities to compensate. In addition to previously mentioned limitations, MALDI-MSI ion images are not directly indicative of the histological setting, as immunohistochemistry (IHC) could be. Consequently, MALDI-MSI will tend to move toward multimodal imaging which can compensate for these limitations by combining chemical and histological information from various modalities, and which results in a more informative dataset.

Multimodal imaging can be used for guided acquisitions. For instance, the acquisition of MALDI-MS images can be guided with microscopy images, so as to restrict the imaged area and reduce the acquisition time and data size (Patterson et al., 2018a; Blanc et al., 2018). Rabe et al. (2018) guide the MALDI-MSI acquisition by performing a segmentation of brain tissue based on Fourier transform infrared spectroscopy (FT-IR), reducing time and data size by nearly 98%.

**FIGURE 1:**
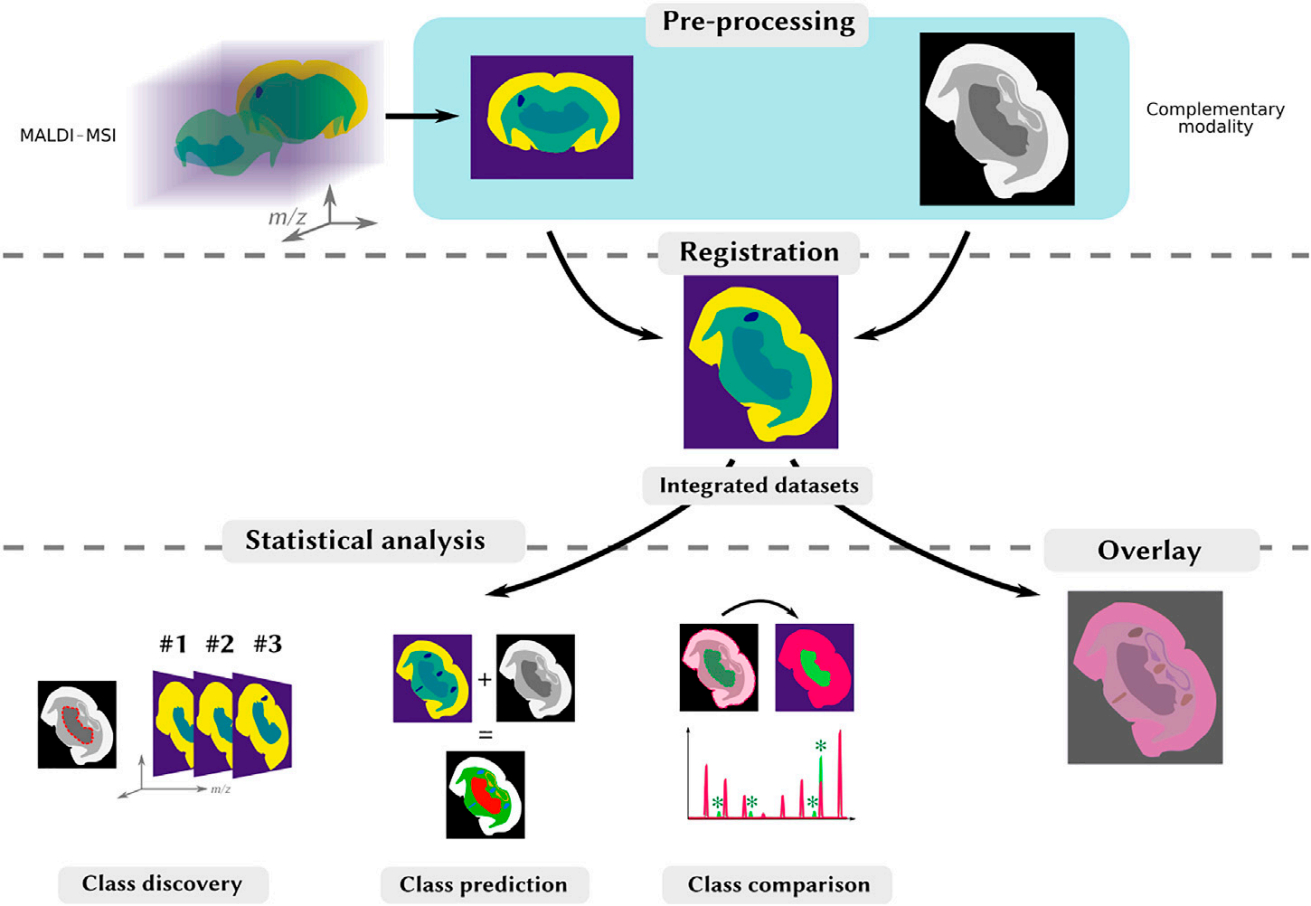
Typical processing and analysis workflow for multimodal MALDI-MSI. Top-row: the MALDI image (on the left), and the complementary images (on the right) are processed so as to have comparable shapes, through various specific processing steps, including segmentation. Middle-row: the images are spatially aligned through registration. Bottom-row: once the images are registered, they can be visualized on-top of each other to reveal similar spatial distributions (on the right). Patterns can be objectified by a joint statistical analysis to, e.g., find spatial correlations, by investigating spatial clusters (class discovery), produce an enriched dataset from both modalities (class prediction) or find region-specific ions in two different ROIs (class comparison).

Multimodal imaging often combines several imaging modalities and yields integrated multimodal datasets, opening new possibilities for data mining. The methods used depend on the complementary modality associated with MALDI-MSI, the processing strategies and scientific goal (Table 1). Integrated multimodal datasets are typically obtained by a three-step-workflow: multimodal acquisition, registration, or the spatial alignment of imaging datasets, and data analysis to highlight correlations between imaging datasets (Figure 1). The objective is to use information from both modalities to evidence complementarities and correlations with different data processing strategies, as described by Bemis et al. (2019): (1) class discovery to find commonalities (spectral or spatial) without a priori information, (2) class prediction, using existing information to infer spatial or biological knowledge in a new dataset and (3) class comparison, which compares intensity averages in regions of interest (ROIs) to find differentially-abundant ions.

**TABLE 1:** Collection of publications that combine MALDI-MSI with another modality. It attempts to classify each paper by its multimodal strategy and scientific goals. The list is separated *in vivo*/*ex vivo* and grouped by the spectral nature of its complementary modality, i.e., whether it is mono-, multi-channel, i.e., spectra with discrete bands, or hyperspectral, i.e., spectra with continuous bands.

	Modality(ies)	Multimodal strategy			Scientific goal			References
		Acquisition(s) and registration method(s)	Data analysis		Result(s)	Omics	Biological background	
			Strategy(ies)	Method(s)				
*In-vivo*	MRI	Adjacent sections, & iconic: linear & non-linear	Class discovery	HSNE, & Pearson correlation	Colocalizing signal (3D)	Proteins	Murine kidney and pancreas, human colorectal cancer, microbial colonies, & human oral carcinoma	Abdelmoula et al. (2019)
	MRI	Adjacent sections, & iconic: linear	Class prediction, & class comparison	Automated anatomical interpretation & Wilcoxon rank test	Brain atlas, & region-specific ions	Proteins	Human brain	Verbeeck et al., (2017)
	MRI	Adjacent sections, & iconic: linear & non-linear	Class prediction	NMF & inverse NMF, & distance in reduced space	Colocalizing signal	Polysaccharides	Plant: wheat grain	Grélard et al., (2021)
	MRI	Adjacent sections, & iconic: linear & non-linear	Class discovery	Bisecting k-means, & Pearson correlation	Colocalizing signal (3D)	Proteins	Murine kidney	Oetjen et al., (2013)
	MRI	Adjacent sections Iconic: linear	Class comparison	Unpaired t-test	Region-specific ions (3D)	Proteins	Murine brain	Sinha et al. (2008)
	MRI	Adjacent sections, & iconic: linear	Overlay visualization	N/a	Integrated dataset (3D)	Proteins	Murine	Attia et al., (2012)
*Ex-vivo*	H&E	Adjacent and same sections, & landmarks: linear	Class prediction	PLS	Pansharpening, & out-of-sample prediction	Lipids, proteins, metabolites, & drugs	Murine brain	Van De Plas et al. (2015)
	H&E	Adjacent sections, & registration not explained	Class comparison, & class prediction	Various multivariate analysis, ROC curve, & Kruskal-Wallis test	Biomarker discovery	Metabolites	Human Urachal Cancer	Neumann et al., (2021)
	H&E	Same section, & landmark	Class prediction	PLS regression	Out-of-sample prediction	Proteins	Murine brain, & kidney	Prentice et al., (2018)
	Autofluorescence Microscopy	Same and adjacent section, landmark, & iconic: Linear & nonlinear	Class comparison	Histogram of average intensities in multiple ROIs	Guided-acquisition. Region-specific ions	Lipids	Murine brain, kidney, spleen & *P. yoelii*-infected livers, human kidney	Patterson et al., (2018a)
	Autofluorescence Microscopy	Same & adjacent section, Landmark, & iconic: Linear and nonlinear	Class discovery	Weighted correlations	Colocalizing signal	Lipids, metabolites	Murine kidney, & brain	Patterson et al., (2018b)
	IHC	Same section, & registration not explained	Class comparison	ROC curves, & Mann Whitney U test	Biomarker discovery	Proteines	Human breast cancer & liver	Rujchanarong et al., (2021)
	IHC	Same section, & landmark	Class comparison	Overlay visualization, & 2D correlation	Integrated dataset	Lipids	Murine brain – Hunter’s disease	Dufresne et al., (2017)
	IF H&E	Same section, & landmark	N/a	Overlay visualization	Region-specific ions	Lipids	Murine brain – Alzheimer’s disease	Kaya et al., (2017b)
	IF	Same sections, & landmark	Class comparison, & class discovery	k-means clustering, & t-test	Integrated dataset	Lipids	Murine brain – Alzheimer’s disease	Kaya et al., (2017a)
	IF	Same cells Registration not explained	Class discovery	Spatial correlation: Euclidean distance, Pearson correlation, & multivariate analysis: K-means clustering	Colocalizing signal	Lipids	Single cells	Nikitina et al., (2020)
	IF	Same section, & landmark: linear	Class comparison, & class discovery	Correlation Network, Spearman rank order correlation, & Mann Whitney U test	Region-specific ions	Metabolites, lipids	Murine pancreas cancer	Prade et al., (2020)
*Ex-vivo*	Imaging Mass Cytometry	Same section, & registration not explained	N/a	Overlay visualization	Integrated dataset	Lipids	Murine Brain, Human tonsil & breast cancer	Yagnik et al., (2021)
	FT-IR	Same section, & registration not explained	Class discovery	Random forest classifier	Integrated dataset, & colocalizing signal	Lipids, carbohydrates, & nucleic acids	*Eisenia fetida*	Ritschar et al., (2022)
	FT-IR	Same section, & Iconic: linear	Class comparison, & Class discovery	k-means clustering, & t-test	Integrated dataset, & guided acquisition	Metabolites, & lipids	Murine brain, & human gastrointestinal stroma tumors	Rabe et al. (2018)
	FT-IR	Same section, iconic	Class comparison, & class prediction	PCA Data Integration/Laplacian Pyramid Sharpening, & ANOVA	Pansharpening, image fusion, & region-specific ions	Lipids, & peptides/proteins	Murine brain	Neumann et al., (2018)
	Raman Spectroscopy	Same section, landmark, & fiducial aided	Class discovery	PCA-PCA correlation	Integrated dataset, & colocalizing signal	Lipids, & peptides/proteins	Cell Spheroids	Ahlf et al., (2014)
	Raman Spectroscopy	Same section, & landmark: linear	Class prediction	PCA on combined dataset, & 2D correlation	Colocalizing signal	Lipids, & peptides/proteins	Murine brain	Ryabchykov et al., (2018)
	Raman Spectroscopy TOF-SIMS	Same section, & landmark: linear	Class prediction	NMF Data fusion	Pansharpening	Metabolites, & lipids	Murine brain	Race et al., (2020)
	LA-ICP	Same & adjacent sections, & iconic: Linear and nonlinear	Class discovery, class comparison	Pearson correlation k-means, Student’s t-test	Colocalizing signal, region-specific ions	Metals, lipids, proteins	Murine spleen, & liver	Castellanos-Garcia et al., (2021)
	TOF-SIMS	Same section, & landmark	Class discovery	Visual comparison	Guided acquisition, & colocalizing signal	Lipids	Human colon cancer	Desbenoit et al., (2018)
	TOF-SIMS	Same section, & inherently registered	N/a	ROI selection	Guided acquisition	Metabolites	Biofilms	Lanni et al., (2014)
	TOF-SIMS Microscopy	Same section, & landmark	Class discovery	Thresholding, granulometry, & visual assessment	Guided acquisition, & colocalizing signals	Lipids	Cells	Comi et al., (2017)
	TOF-SIMS	Same sections, landmark: linear	Class prediction	CCA, & NMF	Pansharpening	Lipids	Murine brain	Borodinov et al., (2020)
	DESI IMC H&E - IF	Same & adjacent sections, & landmark: linear	Class discovery	2D correlation	Colocalizing signal	Drugs	Murine Pancreatic cancer	Strittmatter et al., (2022)
	DESI	Same section, Registration not explained	Class discovery	Visual comparison	Colocalizing signal	Lipids, & proteins	Murine brain, human glioma	Eberlin et al., (2011)
	MALDI-MSI	Adjacent sections, & iconic: linear	Class prediction, class comparison	Multiblock OPLS	Pansharpening, region-specific ions	Lipids, & proteins	Murine hippocampus & Rat prostate	Wehrli et al. (2020)
	MALDI H&E	Same section, & inherently registered	Class comparison	Ion Fold change calculation	Integrated dataset, region-specific ions, & multi-omics	N-glycans, & peptides/proteins	human carcinomas, & tissue microarrays	Heijs et al., (2016)
	LDI	Same section, & Landmark	N/a	Overlay visualization	Integrated dataset, & region-specific ions	Metabolites, Lipids	Murine brain, & lung	Fincher et al., (2020)

### Acquisition Workflow

Biological questions drive the choice of modalities and their implementation. Multimodal acquisitions involve some critical parameters to be considered in terms of analytical strategy: the use of a same sample section or adjacent section, the level of destructiveness of complementary modalities and the order of operations in which the acquisitions occur. Use of same section is advised to establish cellular or subcellular correlations. Adjacent sections are advised when all modalities are destructive, or when it is desirable to keep the sample preparation processes separate. However, the resulting integrated dataset is not reliably interpretable.

The significance of MALDI-MSI sample preparation in multimodal experiments cannot be overstated as there will be consequences for downstream analysis. Due to sample preparation, MALDI-MSI is often conducted after a less destructive imaging modality. In some cases, complementary images can be acquired after MALDI-MSI to facilitate the registration process. Neumann et al. (2018), Patterson et al. (2018b), and Jones et al. (2020) demonstrated multimodal imaging of MALDI-MSI followed by another technique, which is unintuitive in most instances.

### Registration

Registration is usually the first step of multimodal computational workflow. It consists in aligning two or more images, such that the enclosed objects are superimposed.

#### Pre-Processing

Before registration, images should be comparable and have the same number of spatial dimensions. Standardization ensures that the images are in the same intensity range. Images involved in the registration process may come from unprocessed images (e.g., ion image in MALDI) or from segmentation methods, which extract objects of interest in images. For instance, segmentation maps from dimension reduction techniques (e.g*.*, component image), or from clustering, can be used. Segmentation methods are frequently based on pixel intensities or use the geometrical properties of the objects. In a multimodal context, MALDI-MSI segmentation can involve simple thresholding (Anyz et al., 2017), region growing (Grélard et al., 2021), methods assisting in selecting spatially coherent ion images (Alexandrov and Bartels, 2013), or deep learning (Abdelmoula et al., 2022).

#### Methods

Registration methods aim at estimating the transformation which maps a moving, or deformable image onto a fixed, or reference image. If possible, the moving image is chosen as the one with the lowest resolution, so as to preserve the data from the fixed image with higher resolution. MALDI-MSI datasets are large and it is desirable not to interpolate new signal intensities. Thus, MALDI-MSI are generally chosen as the fixed image. The transformation can be rigid (translation and rotation), affine (rigid with scaling, and shearing), projective (affine with perspective) or non-linear. Non-linear transformations account for local deformations in the image. They are advised for adjacent sections and to accurately model the damages during sample preparation.

Registration methods can be classified in two categories, depending on the information they use: landmarks, or pixel intensities (iconic methods).

Landmarks are features in the image that are salient and easily recognizable. Landmarks can be extraneous marks added during sample preparation or during acquisition, or unique features visible in both images. They can be selected automatically or manually. The minimum number of landmarks depends on the transformation model and the dimensionality: for the 2D case, a minimum of three points is necessary for an affine transformation, and up to four for a projective transformation. Manual methods require user intervention in selecting landmarks. They are useful for images with different spatial resolutions, or when it is difficult to find intensity similarities. It is also the preferred method for partial registration, as the registration of an image subset onto an image depicting a larger part of the tissue. Borodinov et al. (2020) registered MALDI images onto a TOF-SIMS image by manually selecting matching pairs of fiducial markers etched by an ion beam. Ryabchykov et al. (2018) registered Raman images onto larger MALDI-MS images by manually selecting common features from PCA component images.

Iconic methods are based on similarities in pixel intensities. Transformation parameters are modified iteratively until a local optimum of a function, called similarity metric, is reached. The similarity metric measures how well two images match after applying a deformation. Typical similarity metrics are the sum of squared intensity differences (SSD), normalized cross-correlation, and mutual information, which estimates the statistical independence of the intensity distributions of the two images. SSD can be applied when both images have the same intensity range and similar contrast. Normalized cross-correlation or mutual information are employed when images have different contrast or dynamic range. Transformation parameters are updated by an optimization algorithm involving the similarity metric. For instance, linear gradient descent updates transformation parameters based on the slope of the metric. The resulting transformation strongly depends on the initialization parameters.

Iconic methods are used in numerous MALDI-MSI registration tasks. Anyz et al. (2017) aligned histological staining (Hematoxylin Eosin, H&E) and MALDI-MS images. Both images are different in terms of pixel intensities, so the authors extract binary masks of the sample. These masks are registered by an affine transformation, with an iterative gradient descent optimization of the SSD metric. Castellanos-Garcia et al. (2021) combined MALDI-MS with LA-ICP-MS images, so as to merge the extensive molecular information with supplemental metals analysis. They select matching ion images manually in order to estimate both linear, and non-linear transformations. Abdelmoula et al. (2019) registered several 2D MALDI slices with a 3D MRI image in order to obtain a volumetric representation of the MALDI dataset. The MALDI image is reduced by the t-SNE algorithm. The corresponding 2D MR image is selected from the 3D volume manually. Then, both linear and non-linear registration methods are applied, using mutual information as a similarity metric.

Hybrid approaches use both iconic and landmark-based registration methods. Patterson et al. (2018a) registered microscopy and MALDI-MS images, as follows: two microscopy images, before and after MALDI-MSI, were acquired. As they are the same modality, the authors use iconic approaches to register them. The microscopy images are then registered onto an upsampled version of the MALDI-MS image using laser ablation marks as landmarks. This workflow avoids introducing deformation artifacts in the MSI datasets, and preserves the resolution of microscopy images.

The quality of the registration determines the reliability of the results of the subsequent data analysis step. Registration evaluation can be achieved through various metrics, such as the Dice coefficient or the F-measure, which both estimate the proportion of overlapping pixels between the deformed and fixed images. When using non-linear transformations, it is important to select parameters such as to obtain a compromise between shape-matching and intensity fidelity. For instance, Grélard et al. (2021) estimated the shape matching by F-measure, and the intensity fidelity by computing the mutual information between the images before and after applying non-linear registration.

### Data Analysis

The last step of a multimodal workflow is usually the joint analysis of the images. This involves using information from both modalities to evidence complementarities and correlations. The algorithms depend on the scientific goal and can be classified in three classes, as described previously: (1) class discovery, (2) class prediction, and (3) class comparison.

Class discovery finds spatial or spectral similarities in an image, and does not require supplementary information from a complementary modality. It can be achieved by data dimension reduction methods or clustering mentioned previously. Abdelmoula et al. (2019) found segmentation maps of registered MALDI-MS images by a hierarchical variant of the t-SNE algorithm. Then, spatial correlations are established by Pearson correlation coefficient between the segmentation map and the ion images. Ryabchykov et al. (2018) found spatial correlations between Raman and MALDI-MS images by analyzing PCA component images and loadings. This analysis highlights changes in lipid distributions.

Class prediction consists in inferring spatial or spectral information, by using extraneous information which usually come from expert annotations or from regions found in the complementary modality. Borodinov et al. (2020) sharpened MALDI-MS images, i.e., they enhance their spatial resolution, by combining them with TOF-SIMS images. They used Canonical Correlation Analysis (CCA) on NMF component images to find similarities. This decomposition and transformation enact the reconstruction of MALDI ion images from the high-resolution TOF-SIMS images. van de Plas et al. (2015) sharpened MALDI-MS images by microscopy images using Partial Least Squares (PLS) regression. They used these images to find anatomical distributions of ions in the sample, as well as predict distributions out of the sample. Prentice et al. (2018) used out-of-sample prediction to correlate microscopy to MALDI-MSI when the acquisition is not practically obtainable. Wehrli et al. (2020) combined Raman and MALDI-MS images and extract a high-resolution MALDI-MS image. Since both images are hyperspectral, they use a multiblock method derived from Orthogonal PLS.

Class comparison consists in comparing two or more ROIs in an image to highlight patterns that have different intensity distributions across the ROIs. This can typically be achieved by univariate analysis. Verbeeck et al. (2017) automatically identified the spatial distributions observed in registered ion images as a combination of the anatomical structures described in the atlas built from MR images. They then used Wilcoxon rank test to find differently expressed ions between healthy and diseased brain hemispheres. Patterson et al. (2018a) found spatial correlations between two MS datasets by weighing abundance correlations by their degree of pixel overlap. Bemis et al. (2019) proposed several models based on hierarchical Bayesian spatial models to find differently abundant ions. They modeled the fact that neighboring pixels have similar spectra, which retrieves differently abundant ions that are missed by other models.

Class discovery, prediction and comparison can be integrated in the same workflow. Jones et al. (2019) used various methods encompassing all these analyses. They combined MALDI-MS images with various microscopy images (fluorescence and H&E). First, they manually selected ions that visibly colocalize with ROIs in the tissue. This approach was insufficient as it led to subjective results, and was not tractable on large datasets. They performed class discovery by the spatially shrunken centroid clustering algorithm and analyzed the clustering results further by looking up ions that were representative of a cluster of interest. Class prediction achieved by PLS regression, which allows to approximate MALDI ion images from a linear combination of fluorescence microscopy channels. The resulting regression made it easy to find biomarkers that were specifically distributed in a specific fluorescence channel. Finally, they performed class comparison on weighted averages in ROIs by Student t-test.

## Perspectives

Multimodal imaging strategies involving MALDI-MSI alleviate technological issues related to scientific questions, leading to a better understanding of the living. It offers new opportunities in biology by allowing direct correlation between cellular and molecular information, but requires diligent approaches for acquisition, registration and computational data analysis to combine and get the best of the different modalities. Once achieved, these combinations raise new questions and initiate new studies, which would not have been possible with a single modality as shown in Table 1.

After highlighting relevant methodological developments, multimodal imaging better contributes or provides evidence towards the biological understanding. Grélard et al. (2021) correlated water distribution given by MR images with the distribution of specific polysaccharides in MS images. This study hints at the role of these molecules in the cell-wall porosity on tissue, and corroborates previous observations made *in vitro*. Strittmatter et al. (2022) combined MALDI and DESI to study the distribution of a drug and its different metabolites, which cannot be established by a single modality. Then, they combined MSI and IMC to show that drug metabolites co-localize with immunohistochemistry markers for DNA damage. Prade et al. (2020) correlated multiplexed fluorescent immunohistochemical staining with MS images to identify ions involved in metabolic networks. They showed discrepancies in metabolic distributions according to different cell populations.

The capabilities achieved by multimodal imaging workflows are numerous, and many are yet to be fully exploited. The technical progress enacting higher spatial resolution for MALDI-MS images is met with larger dataset sizes. These datasets do not necessarily fit in memory, thus memory requirements become a bottleneck to several research groups. Bemis and Vitek (2017) developed an R package which loads larger-than-memory datasets, using on-disk data structures. This effort should be furthered in other programming languages as well. This involves adapting the existing computational methods such that it complies with these data structures, which would require significant work and collaboration from the community.

The variety of processing algorithms makes it difficult to pick suitable methods. Deep learning could facilitate the processing and joint analysis of MS datasets. Abdelmoula et al. (2022) used a deep learning architecture to classify tumor and healthy pixels in MS datasets. One of the benefits of this approach is that it does not rely on spectral and signal processing. More generally, it could facilitate the interpretation of MSI datasets and their integration with other modalities.

What is clear, however, is that we are at the beginning of an exciting new era of analytical chemistry and biomedical research. Groups have laid the groundwork for what we hope will lead to new understandings, new discoveries and new kinds of questions in medicine and biochemistry. And, in turn, these new questions will be met with the emergence of new technologies.
